# Increased risks of retinal vascular occlusion in patients with migraine and the protective effects of migraine treatment: a population-based retrospective cohort study

**DOI:** 10.1038/s41598-024-66363-9

**Published:** 2024-07-04

**Authors:** Kuan-Yun Ho, Chia-Der Lin, Tzu-Ju Hsu, Yu-Han Huang, Fuu-Jen Tsai, Chiao-Ying Liang

**Affiliations:** 1 Department of Medical Education, Taichung Veterans General Hospital, Taichung, Taiwan; 2 Department of Ophthalmology, China Medical University Hospital, China Medical University, Taichung, Taiwan; 3 Department of Otorhinolaryngology-Head and Neck Surgery, China Medical University Hospital, Taichung, Taiwan; 4 School of Medicine, China Medical University, Taichung, Taiwan; 5 Management Office for Health Data (DryLab), Clinical Trial Research Center (CTC), China Medical University Hospital, Taichung, Taiwan; 6 School of Chinese Medicine, College of Chinese Medicine, China Medical University, Taichung, Taiwan; 7 Department of Medical Research, China Medical University Hospital, Taichung, Taiwan; 8 Department of Ophthalmology, Taichung Veterans General Hospital, Taichung, Taiwan

**Keywords:** Migraine, Retinal vascular occlusion, Epidemiology, Blindness, Neurology, Risk factors

## Abstract

Associations between migraine and retinal vascular occlusion have been reported, but there is no large-scale and comprehensive study. Therefore, we aimed to determine risks of retinal vascular occlusion in patients with migraine. Using the Taiwan National Health Insurance Research Database from 2009 to 2020, we enrolled 628,760 patients with migraine and 628,760 matched individuals without migraine. Study outcomes were diagnoses of retinal vascular occlusion, including retinal artery occlusion (RAO) and retinal vein occlusion (RVO). Adjusted hazard ratio (aHR) of retinal vascular occlusion related to migraine was estimated. The cumulative incidences of subsequent retinal vascular occlusion, RAO, and RVO were significantly higher in migraine patients compared with controls (0.31% vs. 0.21%; 0.09% vs. 0.05%; 0.22% vs. 0.17%; all p < 0.001). The hazards of retinal vascular occlusion, RAO, and RVO were significantly greater in the migraine group (aHR, 1.69 [95% CI, 1.57, 1.83], 2.13 [95% CI, 1.84, 2.48] and 1.53 [95% CI, 1.40, 1.68], respectively). Risks of retinal vascular occlusion were significantly higher in migraine both with aura (MA) and without aura (MO) (aHR, 1.77 [95% CI, 1.58, 1.98], and 1.92 [95% CI, 1.64, 2.25]). Among patients with migraine, nonsteroidal anti-inflammatory drugs, propranolol, and flunarizine significantly reduce their risks of retinal vascular occlusion (aHR, 0.19 [95% CI, 0.16, 0.22], 0.73 [95% CI, 0.62, 0.86], 0.84 [95% CI, 0.76, 0.93]). Migraine, MA and MO are independently associated with higher risks of retinal vascular occlusion, RAO, and RVO.

## Introduction

Retinal vascular occlusion, including retinal artery occlusion (RAO) and retinal vein occlusion (RVO), is a leading cause of sudden and painless sight loss. RAO, with a yearly incidence of 1 to 2 cases per 100,000 people, is considered as an ocular analogue of cerebral stroke^1^. It is mostly due to atherosclerosis-related embolism, with carotid artery disease being the main source embolism^2^. In comparison, RVO is more common, with a global prevalence of ~ 0.5%^3^. The pathogenesis of RVO presumably follows the principle of Virchow’s triad for thrombogenesis, i.e., vessel damage, stasis, and hypercoagulability^4^. The main cause of thrombogenesis is due to atherosclerosis of the retinal artery, which further compresses the retinal vein in the lamina cribrosa, secondarily inducing thrombosis in the vein. RAO is categorized into central RAO (CRAO) and branch RAO (BRAO), depending on the occluded artery. The duration of occlusion is further distinguished as permanent, including CRAO and BRAO, and transient, i.e., transient RAO (TRAO). Classification of RVO can be broken down into central RVO (CRVO) and branch RVO (BRVO), based on whether the thrombosis is within the central retinal vein or a branch retinal vein^5^.

Migraine is a common and disabling disorder, affecting ~ 15% of the global population. It is ranked as the 3rd most common disease, and the 7th highest specific cause of disability worldwide^6^. During a migraine attack, the trigeminovascular system has a key role to play. Cerebrovascular disorders, either ischemic stroke or hemorrhagic stroke, and other cardiovascular diseases are more prevalent in migraine patients^7^.

Since migraine and retinal vascular occlusion share similar neurovascular characteristics, migraine is known to associate with retinal vascular occlusion. The evidence is mostly based on case reports on the association between migraine and RAO^8–12^. Few studies have been reported on the association between migraine and RVO^13–15^. There is yet no large-scale and comprehensive study on the association between migraine and retinal vascular occlusion. Whether migraine treatment might affect the risks of retinal vascular occlusion in migraine patients remains unclear.

We hypothesized that patients with migraine may have a higher risk of incident retinal vascular occlusion, RAO, and RVO. Here, by using a large-scale population-based cohort for our study, we aimed to determine the association between migraine and retinal vascular occlusion, and the potential effects of migraine treatment on such risk.

## Methods

### Study design and data source

We performed a retrospective cohort study of a nationwide population with subjects aged > 20 years old. The data were retrieved from the Taiwan National Health Insurance Research Database (NHIRD) covering a period of 12 years from January 1, 2009, to December 31, 2020. NHIRD, which is maintained by the National Health Research Institutes (NHRI) of Taiwan, contains comprehensive medical and pharmacy records on medical care settings for > 99% of the 23 million Taiwanese residents. The database is generated under compulsory and universal national health insurance. Diagnoses are registered using the ICD-9-CM (International Classification of Diseases, Ninth Revision, Clinical Modification; 2009–2015) and ICD-10-CM (International Classification of Diseases, Tenth Revision, Clinical Modification; 2016–2020). For patient privacy, the NHRI has encrypted names of patients, health care providers, and medical institutions with unique and anonymous identifiers prior to releasing the database for research purposes. Consequently, according to the rules of the Institutional Review Board, our study protocol was exempt from an informed consent requirement. Our study was approved by the ethical committee of China Medical University Hospital (CMUH111-REC2-109(CR-1)). All methods were performed in accordance with relevant guidelines and regulations.

### Criteria of inclusion and exclusion

**Figure 1:**
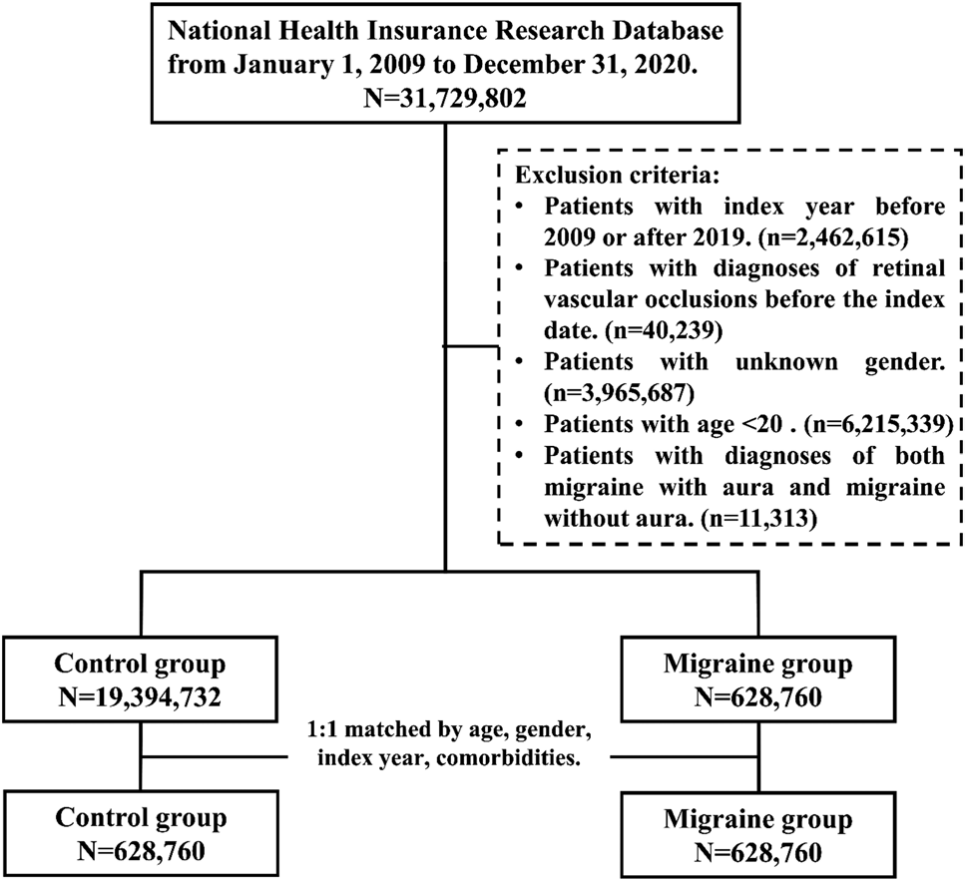
Flow diagram of study sample selection. Index year was the year of index date. The definition of index date for the migraine group was the date of their initial diagnosis during January 1, 2009, to December 31, 2019, whereas index date for the control group was the date of enrollment.

In the migraine group, the diagnosis of migraine was defined as at least 3 outpatient records or one inpatient claim of a migraine diagnostic code made by a neurologist. The definition of index date for a migraine patient was the date of their initial diagnosis within the period from January 1, 2009 to December 31, 2019. Patients were excluded if they had any of the following conditions: (a) diagnosed with retinal vascular occlusion before the index date; (b) unknown gender; and (c) age < 20 years. Individuals without a baseline history of migraine, and who were not diagnosed with migraine during the follow-up period served as the control group. The index date of a control subject was the date of enrollment. To address the bias caused by group differences, the migraine group and the control group were matched at 1:1 ratio, based on propensity score (PS). PS was generated based on age, gender, index year (the year of index date), and comorbidity. We included comorbidities likely associated with RAO or RVO, and further categorized these comorbidities into various groups: i.e., vascular risk factors, thrombotic and embolic diseases, cardiovascular diseases, inflammation in or around the vessel wall, and other diseases (Supplementary Table S1). Those migraine patients who were not able to be matched with non-migraine controls were all excluded. Diagnosis codes we used are shown in Supplementary Table S1. Study outcomes were diagnoses of retinal vascular occlusion, i.e., RAO and RVO. Patients were followed up to determine their incidence of retinal vascular occlusion until December 31, 2020, or death, whichever came first. Figure 1 shows the flow chart.

### Statistical analyses

This is the primary analysis of the data. No statistical power calculation was conducted prior to the study, and the sample size was based on the available data. Considering the large sample size of our study population, demographic characteristics and comorbidities were compared between the migraine and control groups using standardized mean difference (SMD) for both continuous and categorical variables. Any SMD value < 0.1 was considered to reflect no difference between the two groups. The Kaplan–Meier method was used to determine the cumulative incidence of subsequent retinal vascular occlusion in both the migraine and non-migraine cohorts, and results analyzed with the log-rank test. The univariate Cox regression analysis was used to estimate the crude hazard ratios (cHRs) for retinal vascular occlusion related to migraine. The multivariable Cox proportional regression model was used to calculate the adjusted hazard ratios (aHRs) after covariate adjustment for age, gender, comorbidities, and medications. The analyses of Schoenfeld residuals were used to test the assumptions of our Cox proportional hazards model, and the results indicated that using this model for analysis is appropriate. (Retinal vascular occlusion: p = 0.31; RAO: p = 0.72; RVO: p = 0.09) (Supplementary Fig. S1).

In addition, we calculated separately aHRs for RAO and RVO, together with their subtypes: i.e., CRAO, BRAO, TRAO, CRVO, and BRVO. To further determine age effects on risks of retinal vascular occlusion, and gender, we stratified ages into 4 groups (20–40; 40–60; 60–80; and > 80 years); and similarly gender effects (male vs female). In addition, given the wide range of time followed in the study, multivariable analysis was adjusted for the index year (follow-up time of less than 3 years, 3 to 5 years, and more than 5 years). The aHRs of retinal vascular occlusion among migraine subtypes, including migraine with aura (MA) and migraine without aura (MO), were also calculated. The diagnostic codes of these subtypes are shown in Supplementary Table S1.

We further studied effects of different migraine medications on the risk of retinal vascular occlusion. For the definition of treatment for migraine, we only included specific migraine medications which were advised by American Headache Society and European treatment guideline for acute and preventive treatment of migraine. The choice of treatment for acute migraine attacks included nonsteroidal anti-inflammatory drugs (NSAIDs), triptans, and ergotamine. Preventive medications involved propranolol, metoprolol, topiramate, valproate, and flunarizine^16,17^. The corresponding pharmaceutical codes based on Anatomical Therapeutic Chemical (ATC) Classification System for each subgroup and details on the specific NSAIDs included in our study are shown in Supplementary Table S2. To make sure of a causal relationship, we only included migraine medications which had been prescribed after the first diagnosis of migraine, and prior to the study outcomes. Also, only those medications which have been prescribed for a least 2 times were included.

All statistical operations were performed using the SAS statistical software, version 9.4 (SAS Institute, Cary, NC, USA). Graphs were plotted with RStudio. Data were presented as mean ± standard deviation (SD), and 95% confidence intervals (CIs) were provided when appropriate. Two-tailed t-tests were conducted for analyses and p-value less than 0.05 was considered significant.

## Results

### Demographic data

A total of 1,257,520 participants were enrolled in the study, including 628,760 in the migraine group and 628,760 PS-matched controls. The mean age of all participants was 45.35 ± 14.86 years, with 27.07% men and 72.93% women. Table 1 shows the demographic characteristics of the two groups. The mean following period was 7.43 (± 3.21) and 6.77 (± 3.49) years, respectively, in the migraine and control cohort. Since the two groups were well-matched in PS, we therefore found no inter-group difference in terms of age and comorbidities (SMD < 0.1 for each covariate).

**Table 1:** Characteristics of the study subjects.

	Total (n = 1,257,520)	Migraine group (n = 628,760)	Control group (n = 628,760)	SMD
Migraine subtypes, n (%)				
Migraine with aura	–	56,574 (9.00)	–	–
Migraine without aura	–	188,230 (29.94)	–	–
Other subtypes	–	383,956 (61.07)	–	–
Gender, n (%)				
Female	917,083 (72.93)	472,848 (75.20)	444,235 (70.65)	0.103
Male	340,437 (27.07)	155,912 (24.80)	184,525 (29.35)	–
Age, year (mean ± SD)	45.35 ± 14.86	45.32 ± 14.83	45.38 ± 14.90	0.004
20–40, n (%)	526,905 (41.90)	263,924 (41.98)	262,981 (41.83)	0.003
40–60, n (%)	525,600 (41.80)	262,837 (41.80)	262,763 (41.79)	< 0.01
60–80, n (%)	184,561 (14.68)	91,996 (14.63)	92,565 (14.72)	0.003
≥ 80, n (%)	20,454 (1.63)	10,003 (1.59)	10,451 (1.66)	0.006
Comorbidities, n (%)				
Hypertension	263,953 (20.99)	131,838 (20.97)	132,115 (21.01)	0.001
Hypotension	6064 (0.48)	3141 (0.50)	2923 (0.46)	0.005
Diabetes	109,858 (8.74)	54,846 (8.72)	55,012 (8.75)	0.001
Hyperlipidemia	234,912 (18.68)	117,720 (18.72)	117,192 (18.64)	0.002
Stroke	66,408 (5.28)	33,332 (5.30)	33,076 (5.26)	0.002
Pulmonary embolism	470 (0.04)	270 (0.04)	200 (0.03)	0.006
Deep vein thrombosis	2271 (0.18)	1181 (0.19)	1090 (0.17)	0.003
PAOD	6291 (0.50)	3227 (0.51)	3064 (0.49)	0.004
Congestive heart failure	17,103 (1.36)	8513 (1.35)	8590 (1.37)	0.001
Coronary artery disease	101,237 (8.05)	50,770 (8.07)	50,467 (8.03)	0.002
Valvular heart disease	47,277 (3.76)	24,136 (3.84)	23,141 (3.68)	0.008
Atrial fibrillation	6643 (0.53)	3285 (0.52)	3358 (0.53)	0.002
SLE	3801 (0.30)	1996 (0.32)	1805 (0.29)	0.006
Systemic vasculitis	1297 (0.10)	689 (0.11)	608 (0.10)	0.004
Retinal vasculitis	81 (0.01)	51 (0.01)	30 (0.00)	0.004
Glaucoma	33,399 (2.66)	17,003 (2.70)	16,396 (2.61)	0.006
Chronic kidney disease	46,347 (3.69)	23,106 (3.67)	23,241 (3.70)	0.001
COPD	200,681 (15.96)	101,978 (16.22)	98,703 (15.70)	0.014
Obesity	12,913 (1.03)	6707 (1.07)	6206 (0.99)	0.008
Smoking	15,532 (1.24)	7970 (1.27)	7562 (1.20)	0.006
Alcoholism	10,071 (0.80)	5136 (0.82)	4935 (0.78)	0.004
Medication, n (%)				
Acute treatment				
NSAIDs	1,129,070 (89.79)	602,239 (95.78)	526,831 (83.79)	0.404
Triptans	26,139 (2.08)	25,933 (4.12)	206 (0.03)	0.290
Ergotamine	186,345 (14.82)	171,415 (27.26)	14,930 (2.37)	0.748
Preventive treatment				
Propranolol	242,696 (19.30)	205,040 (32.61)	37,656 (5.99)	0.717
Metoprolol	7659 (0.61)	5042 (0.80)	2617 (0.42)	0.050
Topiramate	32,986 (2.62)	31,393 (4.99)	1593 (0.25)	0.300
Valproate	39,380 (3.13)	28,188 (4.48)	11,192 (1.78)	0.156
Flunarizine	284,549 (22.63)	227,566 (36.19)	56,983 (9.06)	0.685
Follow-up time, years (mean ± SD)	7.09 ± 3.37	7.43 ± 3.21	6.77 ± 3.49	0.197

*SD* standard deviation, *SMD* standardized mean difference, *PAOD* peripheral arterial occlusion disease, *COPD* chronic obstructive pulmonary disease, *SLE* systemic lupus erythematosus.

### Risks of developing retinal vascular occlusion in migraine patients

**Figure 2:**
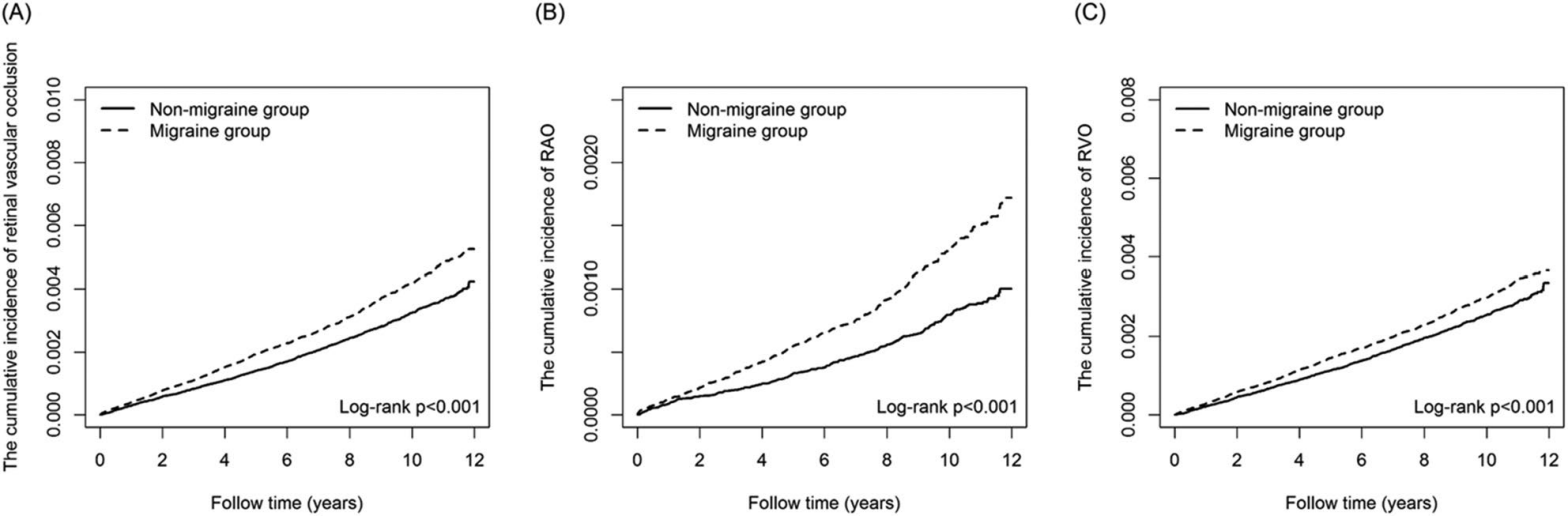
Kaplan–Meier analysis for cumulative incidence of (**A**) retinal vascular occlusion, (**B**) retinal artery occlusion (RAO) and (**C**) retinal vein occlusion (RVO) in patients with and without migraine.

During the 12-year follow-up period, a total of 1921 patients (0.31%) in the migraine group developed retinal vascular occlusion. These cases were 584 RAO (0.09%) and 1389 RVO (0.22%). Whereas in the control group, 1336 (0.21%) patients were diagnosed with retinal vascular occlusion. They were 318 RAO (0.05%) and 1058 RVO (0.17%). Kaplan–Meier curves, after the log-rank test, revealed that the cumulative incidences of subsequent retinal vascular occlusion, RAO, and RVO were significantly higher in migraine patients compared with controls (log-rank test, all p < 0.001) (Fig. 2). The hazard ratios of retinal vascular occlusion during the 12-year study period calculated with univariate and multivariate Cox regression models were displayed in Table 2. Univariate Cox regression analysis showed a significantly higher risk of retinal vascular occlusion in the migraine group, with a cHR of 1.30 (95% CI, 1.22, 1.40; p < 0.001). After adjusting for PS, the risk remained significantly higher in the multivariable regression analysis, with an aHR of 1.69 (95% CI, 1.57, 1.83; p < 0.001). Significant associations with the occurrence of retinal vascular occlusion were found with the following: older age, male, hypertension, diabetes, hyperlipidemia, coronary artery disease, systemic lupus erythematosus (SLE), glaucoma, and chronic kidney disease (CKD) (Table 2).

**Table 2:** Analyses of risk factors for retinal vascular occlusion in patients with migraine in comparison to patients without migraine.

	Univariate analysis		Multivariable analysis	
	cHR (95% CI)	p-value	aHR^a^ (95% CI)	p-value
Migraine (Yes vs. No)	1.30 (1.22, 1.40)	< 0.001	1.69 (1.57, 1.83)	< 0.001
Gender (Male vs. Female)	1.34 (1.25, 1.45)	< 0.001	1.15 (1.07, 1.24)	< 0.001
Age, years				
20–40	Reference	–	Reference	–
40–60	3.86 (3.44, 4.32)	< 0.001	3.17 (2.83, 3.56)	< 0.001
60–80	10.70 (9.55, 12.04)	< 0.001	6.15 (5.41, 7.00)	< 0.001
≥ 80	10.80 (8.72, 13.61)	< 0.001	4.91 (3.88, 6.21)	< 0.001
Comorbidities				
Hypertension	3.83 (3.58, 4.11)	< 0.001	1.92 (1.76, 2.09)	< 0.001
Hypotension	0.77 (0.40, 1.49)	0.443	0.54 (0.28, 1.05)	0.069
Diabetes	2.95 (2.70, 3.22)	< 0.001	1.16 (1.05, 1.28)	0.004
Hyperlipidemia	2.53 (2.35, 2.72)	< 0.001	1.10 (1.01, 1.20)	0.032
Stroke	2.91 (2.61, 3.24)	< 0.001	1.12 (1.00, 1.27)	0.051
Pulmonary embolism	2.48 (0.62, 9.87)	0.197	1.08 (0.27, 4.36)	0.913
Deep vein thrombosis	3.30 (1.91, 5.68)	< 0.001	1.39 (0.80, 2.41)	0.240
PAOD	2.09 (1.44, 3.04)	< 0.001	0.81 (0.56, 1.18)	0.277
Comngestive heart failure	2.66 (2.13, 3.33)	< 0.001	0.84 (0.66, 1.06)	0.137
Coronary artery disease	3.13 (2.86, 3.42)	< 0.001	1.21 (1.09, 1.34)	< 0.001
Valvular heart disease	1.49 (1.26, 1.76)	< 0.001	0.96 (0.81, 1.14)	0.621
Atrial fibrillation	3.61 (2.64, 4.93)	< 0.001	1.22 (0.88, 1.68)	0.226
SLE	1.73 (1.02, 2.92)	0.041	1.83 (1.08, 3.10)	0.025
Systemic vasculitis	2.61 (1.24, 5.48)	0.011	2.05 (0.97, 4.31)	0.060
Retinal vasculitis	6.40 (0.91, 44.83)	0.062	3.18 (0.45, 22.73)	0.248
Glaucoma	3.63 (3.18, 4.15)	< 0.001	1.92 (1.68, 2.20)	< 0.001
Chronic kidney disease	3.34 (2.94, 3.79)	< 0.001	1.38 (1.21, 1.58)	< 0.001
COPD	1.72 (1.58, 1.87)	< 0.001	1.07 (0.98, 1.17)	0.157
Obesity	1.04 (0.71, 1.51)	0.856	0.96 (0.65, 1.40)	0.819
Smoking	0.94 (0.64, 1.39)	0.763	0.87 (0.59, 1.28)	0.481
Alcoholism	1.06 (0.68, 1.64)	0.797	0.85 (0.55, 1.33)	0.480

*PAOD* peripheral arterial occlusion disease, *COPD* chronic obstructive pulmonary disease, *SLE* systemic lupus erythematosus, *cHR* crude hazard ratio, *aHR* adjusted hazard ratio, *CI* confidence interval.

^a^Adjusted HR estimated by the multivariable Cox proportional regression model including the variables of age, gender, and comorbidities.

### Risks of retinal artery occlusion, retinal vein occlusion and their subtypes in migraine patients

**Figure 3:**
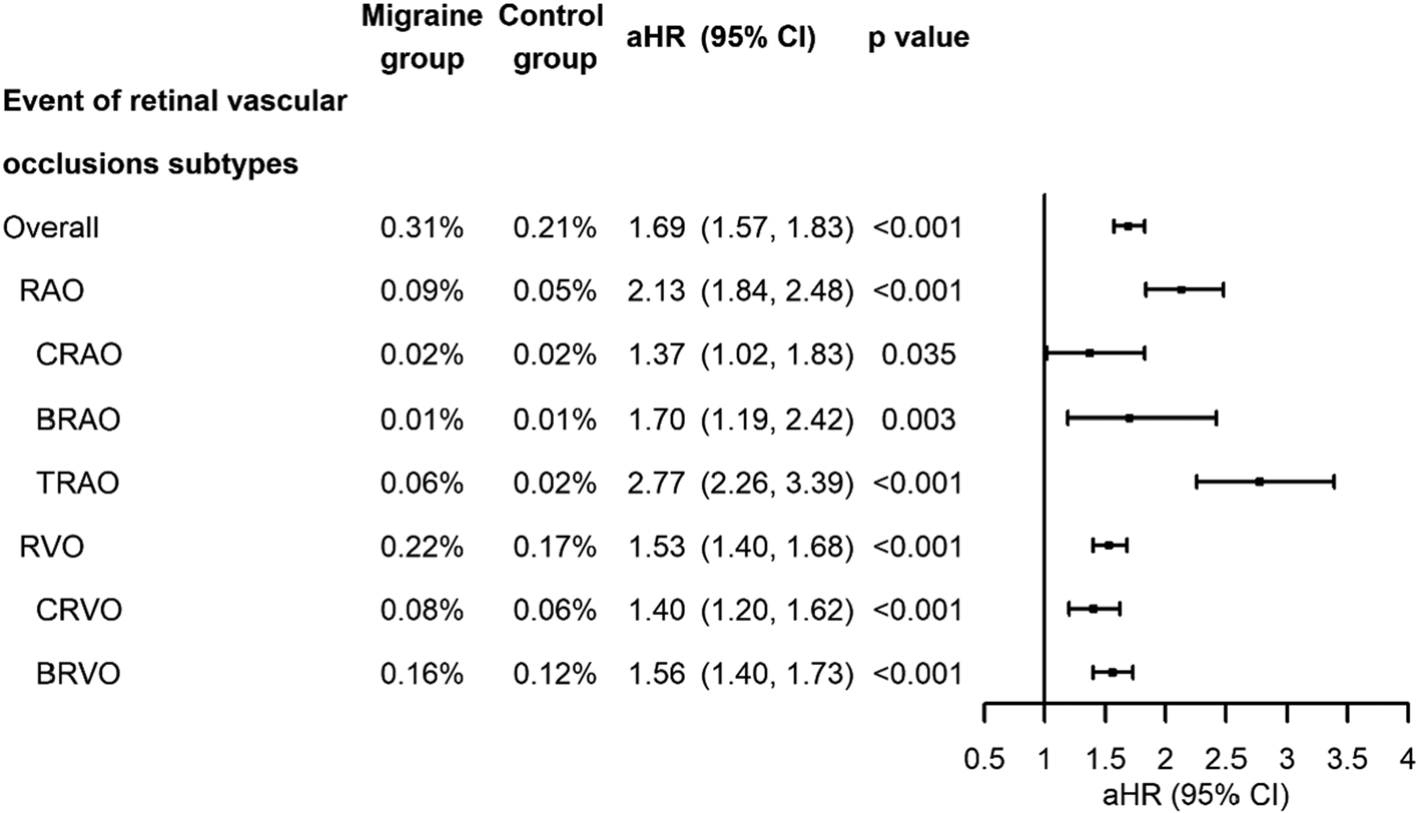
Analyses of the risks of retinal vascular occlusion subtypes in patients with and without migraine. *RAO* retinal artery occlusion, *CRAO* central retinal artery occlusion, *BRAO* branch retinal artery occlusion, *TRAO* transient retinal artery occlusion, *RVO* retinal vein occlusion; *CRVO* central retinal vein occlusion, *BRVO* branch retinal vein occlusion, *aHR* adjusted hazard ratio estimated by the multivariable Cox proportional regression model including the variables of age, gender, and comorbidities, *CI* confidence interval.

The risks of subsequent RAO and RVO in migraine patients were significantly higher (aHR, 2.13 [95% CI, 1.84, 2.48], p < 0.001 and 1.53 [95% CI, 1.40, 1.68], p < 0.001, respectively). Both RAO and RVO shared similar risk factors, i.e., male, older age, hypertension, coronary artery disease, glaucoma, and CKD. Other risk factors associated with RAO were: hyperlipidemia, stroke, SLE, systemic vasculitis, and COPD; while hypotension, diabetes, and congestive heart failure were related with RVO (Supplementary Table S3 and S4). For subgroups of RAO and RVO, we found significant associations between migraine and CRAO (aHR, 1.37 [95% CI, 1.02, 1.83], p = 0.035), BRAO (aHR, 1.70 [95% CI, 1.19, 2.42], p = 0.003), TRAO (aHR, 2.77 [95% CI, 2.26, 3.39], p < 0.001), CRVO (aHR, 1.40 [95% CI, 1.20, 1.62], p < 0.001), and BRVO (aHR, 1.56 [95% CI, 1.40, 1.73], p < 0.001) (Fig. 3).

### Impacts of gender, age, and migraine status on retinal vascular occlusion

In each gender group, the migraine group had a higher aHR of developing retinal vascular occlusion than the control group (female—aHR, 1.64 [95% CI, 1.50, 1.80], p < 0.001; male—aHR, 1.84 [95% CI, 1.60, 2.13], p < 0.001). Additionally, this trend was observed across all age groups. Patients aged 20 to 40 years old and above 80 years old had higher risk of retinal vascular occlusion than other strata (age 20–40–aHR, 2.04 [95% CI, 1.62, 2.57], p < 0.001; age 40–60–aHR, 1.54 [95% CI, 1.37, 1.72], p < 0.001; age 60–80—aHR, 1.80 [95% CI, 1.59, 2.04], p < 0.001; age ≥ 80–aHR, 2.05 [95% CI, 1.28, 3.27], p = 0.003) (Table 3). The higher risks of subsequent RAO and RVO remained significant for both genders and across age strata, except for those aged ≥ 80 (Supplementary Table S5). For the multivariable analysis for the index year, patients with migraine had increased risks of subsequent retinal vascular occlusion, RAO and RVO throughout all follow-up time strata, with the risks highest in the first 3 years following migraine diagnosis. As time progressed, the risks were attenuated, but still statistically significant (Table 4).

**Table 3:** Gender and age stratum and risk for retinal vascular occlusion in patients with migraine in comparison to patients without migraine.

	Univariate analysis		Multivariable analysis	
	cHR (95% CI)	p-value	aHR^a^ (95% CI)	p-value
Female	1.24 (1.14, 1.35)	< 0.001	1.64 (1.50, 1.80)	< 0.001
Male	1.47 (1.29, 1.68)	< 0.001	1.84 (1.60, 2.13)	< 0.001
Age, 20–40	1.58 (1.27, 1.95)	< 0.001	2.04 (1.62, 2.57)	< 0.001
Age, 40–60	1.23 (1.11, 1.36)	< 0.001	1.54 (1.37, 1.72)	< 0.001
Age, 60–80	1.32 (1.18, 1.48)	< 0.001	1.80 (1.59, 2.04)	< 0.001
Age, ≥ 80	1.57 (1.03, 2.40)	0.034	2.05 (1.28, 3.27)	0.003

*cHR* crude hazard ratio, *aHR* adjusted hazard ratio, *CI* confidence interval.

^a^Adjusted HR estimated by the multivariable Cox proportional regression model including the variables of age, gender, and comorbidities.

**Table 4:** Analyses of risks for retinal vascular occlusion in patients with migraine in comparison to patients without migraine with different follow-up time.

	Retinal vascular occlusion		RAO		RVO	
	aHR^a^ (95% CI)	p-value	aHR^a^ (95% CI)	p-value	aHR^a^ (95% CI)	p-value
Migraine (Yes vs. No)						
Follow-up time, years						
< 3	3.17 (2.60, 3.88)	< 0.001	3.32 (2.30, 4.78)	< 0.001	3.01 (2.38, 3.82)	< 0.001
3–5	1.91 (1.63, 2.24)	< 0.001	2.43 (1.73, 3.39)	< 0.001	1.77 (1.48, 2.11)	< 0.001
> 5	1.36 (1.23, 1.50)	< 0.001	1.78 (1.48, 2.13)	< 0.001	1.21 (1.09, 1.36)	< 0.001

*RAO*, retinal artery occlusion, *RVO* retinal vein occlusion, *aHR* adjusted hazard ratio, *CI* confidence interval.

^a^Adjusted HR estimated by the multivariable Cox proportional regression model including the variables of age, gender, and comorbidities.

### Retinal vascular occlusion developments compared between migraine patients with and without aura

**Figure 4:**
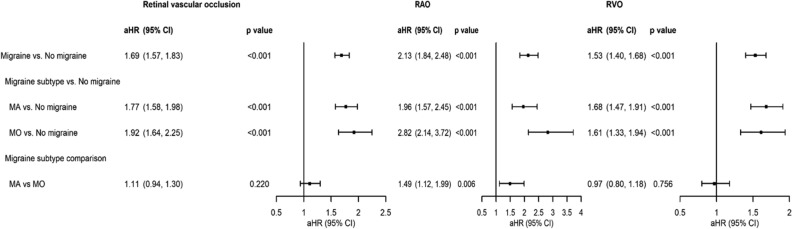
Analyses of the risks of retinal vascular occlusion, retinal artery occlusion (RAO) and retinal vein occlusion (RVO) in the migraine with aura (MA), and migraine without aura (MO) groups. *aHR* adjusted hazard ratio estimated by the multivariable Cox proportional regression model including the variables of age, gender, and comorbidities, *CI* confidence interval.

Risks of subsequent retinal vascular occlusion were significantly higher in patients with MA and MO compared with controls (aHR, 1.77 [95% CI, 1.58, 1.98], p < 0.001; 1.92 [95% CI, 1.64, 2.25], p < 0.001, respectively). The higher risks of developing RAO and RVO remained significant. Compared with MO, MA was associated with a significantly higher risk of RAO (aHR, 1.49 [95% CI, 1.12, 1.99], p = 0.006), but not in retinal vascular occlusion (aHR, 1.11 [95% CI, 0.94, 1.30], p = 0.220) or RVO (aHR, 0.97 [95% CI, 0.80, 1.18], p = 0.757) (Fig. 4).

### Effects of migraine treatment on the risk of retinal vascular occlusion

Risks of retinal vascular occlusion, RAO, and RVO in migraine patients treated with NSAIDs were significantly lower than those without such treatment (aHR, 0.19 [95% CI, 0.16, 0.22], p < 0.001; 0.18 [95% CI, 0.13, 0.23], p < 0.001; 0.21 [95% CI, 0.17, 0.25], p < 0.001, respectively). Propranolol similarly reduced risks of retinal vascular occlusion, RAO, and RVO (aHR, 0.73 [95% CI, 0.62, 0.86], p < 0.001; 0.61 [95% CI, 0.44, 0.83], p = 0.002; 0.77 [95% CI, 0.63, 0.93], p < 0.006). Flunarizine significantly decreased the risks of retinal vascular occlusion and RVO (aHR, 0.84 [95% CI, 0.76, 0.93], p < 0.001; 0.77 [95% CI, 0.68, 0.86], p < 0.001). Triptans, ergotamine, metoprolol, topiramate, and valproate however did not show such effect on reducing the risks of retinal vascular occlusion (aHR, 0.88 [95% CI, 0.64, 1.22], p = 0.454; 1.12 [95% CI, 0.94, 1.33], p = 0.191; 1.15 [95% CI, 0.81, 1.64], p = 0.429; 0.82 [95% CI, 0.62, 1.07], p = 0.147; 0.80 [95% CI, 0.64, 1.01], p = 0.059) (Table 5).

**Table 5:** Analyses of migraine treatment effects on the risks of retinal vascular occlusion.

	Retinal vascular occlusion		RAO		RVO	
	aHR^a^ (95% CI)	p-value	aHR^a^ (95% CI)	p-value	aHR^a^ (95% CI)	p-value
Migraine patients with vs without medications						
NSAIDs						
Without	Reference	–	Reference	–	Reference	–
With	0.19 (0.16, 0.22)	< 0.001	0.18 (0.13, 0.23)	< 0.001	0.21 (0.17, 0.25)	< 0.001
Triptans						
Without	Reference	–	Reference	–	Reference	–
With	0.88 (0.64, 1.22)	0.454	1.15 (0.71, 1.87)	0.568	0.81 (0.54, 1.22)	0.314
Ergotamine						
Without	Reference	–	Reference	–	Reference	–
With	1.12 (0.94, 1.33)	0.191	1.14 (0.82, 1.58)	0.438	1.15 (0.94, 1.41)	0.170
Propranolol						
Without	Reference	–	Reference	–	Reference	–
With	0.73 (0.62, 0.86)	< 0.001	0.61 (0.44, 0.83)	0.002	0.77 (0.63, 0.93)	0.006
Metoprolol						
Without	Reference	–	Reference	–	Reference	–
With	1.15 (0.81, 1.64)	0.429	1.59 (0.89, 2.84)	0.114	1.10 (0.73, 1.67)	0.643
Topiramate						
Without	Reference	–	Reference	–	Reference	–
With	0.82 (0.62, 1.07)	0.147	0.72 (0.44, 1.18)	0.194	0.84 (0.61, 1.16)	0.298
Valproate						
Without	Reference	–	Reference	–	Reference	–
With	0.80 (0.64, 1.01)	0.059	0.55 (0.33, 0.89)	0.015	0.89 (0.69, 1.15)	0.373
Flunarizine						
Without	Reference	–	Reference	–	Reference	–
With	0.84 (0.76, 0.93)	< 0.001	1.09 (0.91, 1.30)	0.344	0.77 (0.68, 0.86)	< 0.001

*RAO* retinal artery occlusion, *RVO* retinal vein occlusion, *NSAIDs* Non-steroidal anti-inflammatory drugs, *aHR* adjusted hazard ratio, *CI* confidence interval.

^a^Adjusted HR estimated by the multivariable Cox proportional regression model including the variables of age, gender, and comorbidities.

## Discussion

In this large population study, we determined the associations between migraine and retinal vascular occlusion, including RAO and RVO, as well as the protective effects of migraine treatment, including NSAIDs, propranolol, and flunarizine. Despite the report of Al-Moujahed et al. on the association between migraine and RAO in the US population, no study has yet been reported on the relationship between migraine and RVO, and little is known on the effects of migraine treatment on such relationship^18^. In addition, racial differences are known regarding risk factors of both RAO and RVO^19^. Our study cohort represents the entire Taiwanese population. We found that migraine is associated with higher risks of retinal vascular occlusion, including RAO and RVO, as well as their subtypes.

Al-Moujahed et al.reported a threefold increase of RAO risk in US migraine patients, whereas we found a twofold increase in Taiwanese patients. Our slightly lower risk may be explained as follows. First, giant cell arteritis (GCA), which further causes RAO with an inflammatory etiology, is predominant in Caucasians, but rare in Asians^20^. The symptoms of GCA may mimic migraine, thus leading to misdiagnosis. In our study, there were a total of 589 patients diagnosed with GCA, accounting for 0.05% of the population. Among the group with migraine, there were 548 patients with GCA (0.09%), while in the non-migraine group, there were 48 patients with GCA (0.01%). According to previous studies, the prevalence of GCA was about 0.25% in the United Kingdom and 0.20% in the United States and Canada^21^. Second, the discrepancy in results may be race-related. Since previous meta-analyses and cohort studies reported that the risk of ischemic stroke in Caucasian migraineurs is higher than that in Taiwanese migraineurs^22,23^. We also demonstrated that the risks of retinal vascular occlusion, RAO and RVO were particularly higher at short-term follow-up, with a > threefold increase in the first 3 years following migraine diagnosis. The risks reduced to a < twofold increase at long-term follow-up (i.e., more than 5 years after migraine diagnosis).

Though the mechanisms of migraine on retinal vascular occlusion are likely multifactorial and currently elusive, several hypotheses have been proposed. First, alterations in endothelial and arterial function, which predispose to atherosclerosis and cardiovascular diseases, constitute an important link between migraine and vascular diseases^24^. It has been demonstrated that vascular endothelial dysfunctions are present in migraine patients and retinal vascular occlusion based on studies with biomarkers. For example, a number of ultrasound studies revealed a drop in flow mediated dilation (FMD) in brachial artery^25–27^. Second, atrial fibrillation, which is a leading cause of cardiac emboli, was demonstrated to contribute to retinal vascular occlusion, and also significantly associated with MA^28,29^. Third, mutations of the methylenetetrahydrofolate reductase (MTHFR) gene, including MTHFR C677T and A1298C, affect homocysteine levels, causing hyperhomocysteinemia. Hyperhomocysteinemia is considered to be associated with migraine and retinal vascular occlusion^30,31^. Fourth, there is a connection between migraine and retinal vascular occlusion with hereditary cerebral small vessel diseases, including cerebral autosomal dominant arteriopathy with subcortical infarcts and leukoencephalopathy (CADASIL), and retinal vasculopathy with cerebral leukodystrophy (RVCL). Migraine and stroke are clinical features of both CADASIL and RVCL, whereas vascular retinopathy was featured in patients with RVCL^32^. Fifth, multiple coagulation abnormalities, such as platelet abnormalities and hypercoagulable states, have been detected in migraine patients with retinal vascular occlusion^33,34^. Sixth, migraine patients are at a higher risk of patent foramen ovale (PFO) and carotid arterial dissection, both of which may contribute to RAO. It was hypothesized that intracardiac right-to-left shunting through a PFO, accentuated by Valsalva maneuver, may predispose embolic events^35–38^. Last but not least, during migraine attack, cerebral arterial vasospasm may hypothetically cause abnormal blood flow in the retina, and further contributes to acute retinal ischemia^39^. This hypothesis is supported by the evidence of significantly reduced vessel and perfusion density over macula in migraine patients. RAO is also commonly accompanied by acute cerebral infarctions at presentation^40,41^. The mechanism of vasospasm could explain the higher risk of TRAO we found in our migraine patients. However, since it may be a challenge to distinguish MA from TRAO, the identified percentage of TRAO may be overestimated due to incorrect diagnosis.

We have demonstrated that migraine patients aged 20–40 years old had the higher risk of retinal vascular occlusion than patients aged 40–60 years old or 60–80 years old. Similar to previous studies of migraine and stroke, it is probably in younger individuals that migraine would contribute the most to the risk of retinal vascular occlusion, as compared to later in life when traditional risk factors such as large artery atherosclerosis or atrial fibrillation are more common. Possible mechanisms of migraine and retinal vascular occlusion in younger individuals included vasospasm, MTHFR gene mutation, coagulation abnormalities, and PFO^42^.

Previous studies reported a doubling risk of ischemic stroke in patients with MA, but no clear association between stroke and MO^35^. The Taiwanese population-based cohort study showed similar findings^23^. Specifically, we found that both MA and MO having significantly higher risks of developing retinal vascular occlusion. Compared with MO, MA was associated with a significantly higher risk of RAO but not with retinal vascular occlusion. Since RAO is considered as an ocular analogue of cerebral ischemic stroke, the result of our study was consistent with the previous studies which linked migraine, particularly MA, with increased risk of ischemic stroke^43^.

Currently, there is no medication recommended for primary prevention of ischemic stroke in migraine patients^35^. Our study is the first large-scale analysis on NSAIDs, propranolol, and flunarizine in migraine patients, and we revealed their protective effects on risks of retinal vascular occlusion. These findings imply that active management of migraine could further reduce risks of retinal vascular occlusion. NSAIDs are anti‐inflammatory agents, which primarily inhibit cyclooxygenase, biosynthesis of prostaglandins, as well as cytokines production including Interleukin-1 (IL‐1). As neurogenic inflammation was considered a key factor in the mechanisms of migraine, NSAIDs are the first treatment choice for mild to moderate migraine attacks. Since proinflammatory cytokines such as IL‐1, and their inflammatory reactions are pathophysiological stimuli in endothelial dysfunction and the development of atherosclerosis, both of which contribute to the development of vascular occlusion including stroke and retinal vascular occlusion. Anti-inflammatory drugs may therefore be beneficial in preventing stroke and retinal vascular occlusion^44–46^. On the other hand, previous study showed that there was protective effect of flunarizine on ischemic retinal injury in rat model, and elevated intracellular calcium concentration may play an important role^47^. Both triptans and ergotamine can induce vasoconstriction and potentially increase the risk of serious ischemic events, including stroke^48^. Though the evidence on stroke risk is conflicting in current studies, these potential effects may explain that in our study, triptans and ergotamine have no protective effect on risks of retinal vascular occlusion in migraine patients. Further research is warranted to elucidate the underlying pharmacological mechanisms between migraine treatment and retinal vascular occlusion. Additionally, we found that risk factors of retinal vascular occlusion included hypertension, diabetes, stroke, coronary artery disease, SLE, glaucoma, and CKD. Aggressive management of these diseases may reduce the risk of developing retinal vascular occlusion in migraine patients. Further studies on the specific treatment protocols for these comorbidities in retinal vascular occlusion are needed.

The strengths of our present study are the following: its large case number providing sufficient statistical power, and its cohort study design with a long follow-up period enabling clear time-sequence associations. Confounders including age, gender and comorbidities were adjusted to determine a more accurate association between migraine and retinal vascular occlusion. In addition, our study has thorough analyses of different subtypes of RAO, RVO, and migraine, as well as the migraine treatment effects. The higher risks of retinal vascular occlusion in migraine patients were also demonstrated among all age, gender, and follow-up time strata. However, our study has several limitations. First, in the NHIRD, no data are available on patients’ visual acuity, genetic sequencing, and laboratory data. Second, we cannot rule out the possibility of diagnostic code errors. For example, TRAO may be overestimated because the aura symptoms during MA might be misdiagnosed. For migraine, since it might have been misdiagnosed with other types of headaches, we strictly defined migraine as at least 3 records of a migraine diagnostic code made by a neurologist to make the diagnosis more reliable. Third, some migraine medications may have been prescribed for other indications, resulting in an overestimation of such prescriptions only for migraine. To address this issue, we only included migraine medications which were prescribed after the first diagnosis of migraine and prior to the study outcomes, and only those medications which have been prescribed for a least 2 times were included. On the other hands, some of the medications, such as NSAIDs, are non-prescription drugs, and over-the-counter preparations are available from pharmacies. These medications may be underestimated. Despite the selection bias mentioned above, our study consisted of a large sample size and used PS based on age, gender, index year, and comorbidities, which could potentially minimize the selection bias. Finally, the results of our study, which were representative of the entire Taiwanese population, may not apply to other ethnicities.

In conclusion, this large-scale study has demonstrated that people with migraine have increased risks of developing retinal vascular occlusion, RAO and RVO. The risks of retinal vascular occlusion were significantly higher in patients with MA and MO, and MA carried a significantly higher risk of RAO compared with MO. Among patients with migraine, NSAIDs, propranolol, and flunarizine may have a protective effect on risks of retinal vascular occlusion. Ophthalmologists, neurologists, and physicians should be aware of the possible risk of developing retinal vascular occlusion in migraine patients, and early ophthalmic evaluation and treatment initiation is strongly recommended.

### Supplementary Information

Supplementary Information.

## Data Availability

The datasets used or analyzed during the present study are available from the corresponding author on reasonable request.
