# Supplementary material for: Increased risks of retinal vascular occlusion in patients with migraine and the protective effects of migraine treatment: a population-based retrospective cohort study
Source: Sci Rep. 2024 Jul 4;14:15429. doi: 10.1038/s41598-024-66363-9 (PMC11224338; doi:10.1038/s41598-024-66363-9)
Supplement: Supplementary file 1 — Supplementary Information. [file 41598_2024_66363_MOESM1_ESM.docx]

| **Supplementary Table S1** Diagnosis codes | | | |
| --- | --- | --- | --- |
| **Disease types** | | **ICD-9** | **ICD-10** |
| Retinal vascular occlusion | | | |
| RAO | Central RAO (CRAO) | 362.31 | H34.10-13 |
|  | Branch RAO (BRAO) | 362.32 | H34.231-233, H34.239 |
|  | Partial RAO (PRAO) | 362.33 | H34.211-213, H34.219 |
|  | Transient RAO (TRAO) | 362.34 | G45.3, H34.00-03 |
| RVO | Central RVO (CRVO) | 362.35 | H34.811-813, H34.819 |
|  | Branch RVO (BRVO) | 362.36 | H34.831-833, H34.839 |
| Migraine | | | |
| Overall | | 346 | G43 |
| Migraine with aura | | 346.0 | G43.1, G43.5, G43.6 |
| Migraine without aura | | 346.1 | G43.0, G43.7 |
| Vascular risk factors | | | |
| Hypertension | | 401-405 | I10-13, I15 |
| Hypotension | | 458 | I95 |
| Diabetes | | 250 | E8-11, E13 |
| Hyperlipidemia | | 272 | E71.30, E75, E77-78, E88 |
| Obesity | | 278, 783.1 | E66.01, E66.09, E66.1-2, E66.8-9, E65, E67.0-3, E67.8, E68, R63.5 |
| Smoking | | 305.1, V15.82 | F17.2, Z87.891 |
| Alcoholism | | 291, 303, 305.0, 571.0-3, 790.3, V11.3, V79.1 | F10, K70, R78.0, Z65.8 |
| Thrombotic and embolic diseases | | | |
| Stroke | | 430-438 | I60-63, I65-69, G45-46 |
| Pulmonary embolism | | 415.19 | I26.9, I27.82 |
| Deep vein thrombosis | | 453 | I81-82 |
| PAOD | | 443-444 | I73-74, I79.1, I79.8 |
| Cardiovascular diseases | | | |
| Congestive heart failure | | 428 | I50 |
| Coronary artery disease | | 410-414 | I20-22, I25 |
| Valvular heart disease | | 394-397, 424.0-424.3 | I05-08, I34-37 |
| Atrial fibrillation | | 427.31 | I48.0-2, I48.91 |
| Inflammation in or around the vessel wall | | | |
| SLE | | 710.0 | M32 |
| Systemic vasculitis^a^ | | 443.1, 446.0, 446.2, 446.4, 446.5, 446.7, 136.1 | I73.1, M30.0, M30.2, M30.8, M31.0-1, M31.4-7, M31.30-31,  M35.2 |
| Retinal vasculitis | | 362.18 | H35.06 |
| Other diseases | | | |
| Glaucoma | | 365 | H40, H42 |
| Chronic kidney disease | | 580-588 | N00-08, 14, 15.0, 15.8, 15.9, 16-19, 25-26, E10-11 |
| COPD | | 491, 492, 493.20-22, 496 | J41-44 |
| ICD-9: International Classification of Diseases, Ninth Revision; ICD-10: International Classification of Diseases, Tenth Revision.  RAO: retinal artery occlusion; RVO: retinal vein occlusion; PAOD: peripheral arterial occlusion disease; COPD: chronic obstructive pulmonary disease; SLE: systemic lupus erythematosus.  a: including Thromboangitis obliterans, Polyarteritis nodosa, Goodpasture's syndrome, Granulomatosis with polyangiitis, Giant cell arteritis, Takayasu's disease, Behcet's syndrome. | | | |

| **Supplementary Table S2** Migraine medications and pharmaceutical codes based on Anatomical Therapeutic Chemical (ATC) Classification System | | |
| --- | --- | --- |
|  | **Drug** | **Pharmaceutical codes** |
| Acute treatment of migraine | NSAIDs^a^ | M01A、M02A |
|  | Triptans | N02CC |
|  | Ergotamine | N02CA |
| Preventive treatment of migraine | Propranolol | C07AA05 |
|  | Metoprolol | C07AB02 |
|  | Topiramate | N03AX11 |
|  | Valproate | N03AG01 |
|  | Flunarizine | N07CA03 |
| NSAIDs: Non-steroidal anti-inflammatory drugs.  a: including Aceclofenac, Acemetacin, Alclofenac, Alminoprofen, Bendazac, Benzydamine, Bufexamac, Celecoxib, Chondroitin, Clofezone, Dexketoprofen, Diclofenac, Esflurbiprofen, Etodolac, Etofenamate, Etoricoxib, Felbinac, Fenbufen, Fenoprofen, Fentiazac, Feprazone, Flufenamic acid, Flurbiprofen, Glucosamine, Ibuprofen, Indomethacin, Ketoprofen, Ketorolac, Loxoprofen, Meclofenamic acid, Mefenamic acid, Meloxicam, Mepirizole, Mofebutazone, Nabumetone, Naproxen, Nifenazone, Niflumic acid, Nimesulide, Oxyphenbutazone, Phenylbutazone, Piketoprofen, Piroxicam, Rofecoxib, Sulindac, Suxibuzone, Tenoxicam, Tiaprofenic acid, Tiaramide, and Tolmetin (listed in alphabetical order) | | |

| **Supplementary Table S3** Analyses of risk factors for retinal artery occlusion in patients with migraine in comparison to patients without migraine | | | | |
| --- | --- | --- | --- | --- |
|  | **Univariate analysis** | | **Multivariable analysis** | |
|  | **cHR (95% CI)** | **p-value** | **aHR^a^ (95% CI)** | **p-value** |
| Migraine (Yes vs. No) | 1.66 (1.45, 1.91) | <0.001 | 2.13 (1.84, 2.48) | <0.001 |
| Gender (Male vs. Female) | 1.46 (1.27, 1.68) | <0.001 | 1.28 (1.11, 1.48) | <0.001 |
| Age, years | | | | |
| 20-40 | Reference | - | Reference | - |
| 40-60 | 2.79 (2.32, 3.36) | <0.001 | 2.31 (1.90, 2.79) | <0.001 |
| 60-80 | 6.22 (5.10, 7.58) | <0.001 | 3.37 (2.68, 4.24) | <0.001 |
| ≥80 | 6.52 (4.20, 10.13) | <0.001 | 2.53 (1.59, 4.04) | <0.001 |
| Comorbidities | | | | |
| Hypertension | 2.83 (2.47, 3.24) | <0.001 | 1.29 (1.09, 1.52) | 0.004 |
| Hypotension | 1.92 (0.86, 4.29) | 0.111 | 1.14 (0.51, 2.57) | 0.746 |
| Diabetes | 2.58 (2.17, 3.08) | <0.001 | 1.05 (0.86, 1.28) | 0.650 |
| Hyperlipidemia | 2.61 (2.27, 3.00) | <0.001 | 1.29 (1.09, 1.52) | 0.003 |
| Stroke | 3.62 (2.98, 4.39) | <0.001 | 1.55 (1.25, 1.92) | <0.001 |
| Pulmonary embolism^b^ |  |  |  |  |
| Deep vein thrombosis | 3.74 (1.40, 9.98) | 0.009 | 1.57 (0.58, 4.21) | 0.370 |
| PAOD | 1.64 (0.74, 3.67) | 0.226 | 0.63 (0.28, 1.40) | 0.256 |
| Comngestive heart failure | 3.69 (2.55, 5.35) | <0.001 | 1.12 (0.75, 1.66) | 0.576 |
| Coronary artery disease | 3.51 (2.97, 4.14) | <0.001 | 1.46 (1.20, 1.78) | <0.001 |
| Valvular heart disease | 1.99 (1.50, 2.64) | <0.001 | 1.18 (0.88, 1.59) | 0.263 |
| Atrial fibrillation | 3.66 (2.02, 6.64) | <0.001 | 1.09 (0.59, 2.02) | 0.773 |
| SLE | 2.72 (1.22, 6.08) | 0.014 | 2.54 (1.13, 5.69) | 0.024 |
| Systemic vasculitis | 5.49 (2.06, 14.67) | <0.001 | 3.83 (1.43, 10.29) | 0.008 |
| Retinal vasculitis^b^ | - | - | - | - |
| Glaucoma | 3.82 (2.97, 4.90) | <0.001 | 2.09 (1.62, 2.70) | <0.001 |
| Chronic kidney disease | 3.69 (2.92, 4.67) | <0.001 | 1.55 (1.21, 2.00) | <0.001 |
| COPD | 2.13 (1.82, 2.48) | <0.001 | 1.36 (1.15, 1.60) | <0.001 |
| Obesity | 1.57 (0.86, 2.84) | 0.139 | 1.37 (0.75, 2.50) | 0.302 |
| Smoking | 1.63 (0.92, 2.88) | 0.094 | 1.28 (0.72, 2.28) | 0.404 |
| Alcoholism | 1.57 (0.78, 3.15) | 0.204 | 1.15 (0.57, 2.32) | 0.696 |
| PAOD: peripheral arterial occlusion disease; COPD: chronic obstructive pulmonary disease; SLE: systemic lupus erythematosus.  cHR: crude hazard ratio; aHR: adjusted hazard ratio; CI: confidence interval.  a: Adjusted HR estimated by the multivariable Cox proportional regression model including the variables of age, gender, and comorbidities.  b: n=0. | | | | |

| **Supplementary Table S4** Analyses of risk factors for retinal vein occlusion in patients with migraine in comparison to patients without migraine | | | | |
| --- | --- | --- | --- | --- |
|  | **Univariate analysis** | | **Multivariable analysis** | |
|  | **cHR (95% CI)** | **p-value** | **aHR^a^ (95% CI)** | **p-value** |
| Migraine (Yes vs. No) | 1.19 (1.10, 1.29) | <0.001 | 1.53 (1.40, 1.68) | <0.001 |
| Gender (Male vs. Female) | 1.31 (1.20, 1.43) | <0.001 | 1.11 (1.02, 1.22) | 0.016 |
| Age, years | | | | |
| 20-40 | Reference | - | Reference | - |
| 40-60 | 4.59 (3.98, 5.29) | <0.001 | 3.78 (3.27, 4.36) | <0.001 |
| 60-80 | 13.90 (12.05, 16.05) | <0.001 | 8.14 (6.96, 9.53) | <0.001 |
| ≥80 | 14.20 (11.04, 18.38) | <0.001 | 6.76 (5.16, 8.84) | <0.001 |
| Comorbidities | | | | |
| Hypertension | 4.26 (3.93, 4.61) | <0.001 | 2.15 (1.95, 2.37) | <0.001 |
| Hypotension | 0.34 (0.11, 1.05) | 0.062 | 0.25 (0.08, 0.78) | 0.017 |
| Diabetes | 3.10 (2.81, 3.43) | <0.001 | 1.20 (1.07, 1.35) | 0.001 |
| Hyperlipidemia | 2.49 (2.29, 2.71) | <0.001 | 1.03 (0.93, 1.14) | 0.577 |
| Stroke | 2.71 (2.38, 3.09) | <0.001 | 1.01 (0.88, 1.16) | 0.840 |
| Pulmonary embolism | 3.25 (0.81, 12.98) | 0.095 | 1.52 (0.38, 6.14) | 0.557 |
| Deep vein thrombosis | 3.34 (1.80, 6.21) | <0.001 | 1.41 (0.75, 2.65) | 0.280 |
| PAOD | 2.28 (1.51, 3.43) | <0.001 | 0.88 (0.58, 1.33) | 0.548 |
| Comngestive heart failure | 2.28 (1.73, 3.01) | <0.001 | 0.72 (0.54, 0.97) | 0.028 |
| Coronary artery disease | 3.04 (2.74, 3.37) | <0.001 | 1.15 (1.02, 1.29) | 0.019 |
| Valvular heart disease | 1.28 (1.04, 1.58) | 0.018 | 0.84 (0.68, 1.04) | 0.114 |
| Atrial fibrillation | 3.68 (2.58, 5.25) | <0.001 | 1.30 (0.91, 1.88) | 0.152 |
| SLE | 1.31 (0.65, 2.61) | 0.452 | 1.44 (0.72, 2.89) | 0.303 |
| Systemic vasculitis | 1.97 (0.74, 5.24) | 0.176 | 1.61 (0.60, 4.30) | 0.346 |
| Retinal vasculitis | 8.42 (1.20, 59.00) | 0.032 | 4.59 (0.64, 32.65) | 0.128 |
| Glaucoma | 3.52 (3.02, 4.11) | <0.001 | 1.82 (1.56, 2.14) | <0.001 |
| Chronic kidney disease | 3.25 (2.81, 3.77) | <0.001 | 1.34 (1.15, 1.57) | <0.001 |
| COPD | 1.59 (1.44, 1.76) | <0.001 | 0.97 (0.88, 1.08) | 0.638 |
| Obesity | 0.81 (0.49, 1.32) | 0.392 | 0.77 (0.47, 1.25) | 0.289 |
| Smoking | 0.76 (0.46, 1.24) | 0.273 | 0.75 (0.45, 1.22) | 0.245 |
| Alcoholism | 0.83 (0.47, 1.47) | 0.532 | 0.70 (0.39, 1.23) | 0.212 |
| PAOD: peripheral arterial occlusion disease; COPD: chronic obstructive pulmonary disease; SLE: systemic lupus erythematosus.  cHR: crude hazard ratio; aHR: adjusted hazard ratio; CI: confidence interval.  a: Adjusted HR estimated by the multivariable Cox proportional regression model including the variables of age, gender, and comorbidities. | | | | |

| **Supplementary Table S5** Gender and age stratum and risk for retinal artery occlusion and retinal vein occlusion in patients with migraine in comparison to patients without migraine | | | | |
| --- | --- | --- | --- | --- |
|  | **Univariate analysis** | | **Multivariable analysis** | |
|  | **cHR (95% CI)** | **p-value** | **aHR^a^ (95% CI)** | **p-value** |
| Female |  |  |  |  |
| Retinal vascular occlusion | 1.24 (1.14, 1.35) | <0.001 | 1.64 (1.50, 1.80) | <0.001 |
| RAO | 1.72 (1.45, 2.03) | <0.001 | 2.24 (1.87, 2.69) | <0.001 |
| CRAO | 1.26 (0.90, 1.75) | 0.183 | 1.57 (1.08, 2.28) | 0.017 |
| BRAO | 1.30 (0.87, 1.93) | 0.200 | 1.70 (1.08, 2.65) | 0.021 |
| TRAO | 2.07 (1.66, 2.58) | <0.001 | 2.73 (2.15, 3.46) | <0.001 |
| RVO | 1.11 (1.01, 1.22) | 0.029 | 1.45 (1.31, 1.61) | <0.001 |
| CRVO | 1.05 (0.90, 1.24) | 0.531 | 1.26 (1.05, 1.51) | 0.012 |
| BRVO | 1.13 (1.02, 1.27) | 0.026 | 1.51 (1.33, 1.71) | <0.001 |
| Male |  |  |  |  |
| Retinal vascular occlusion | 1.47 (1.29, 1.68) | <0.001 | 1.84 (1.60, 2.13) | <0.001 |
| RAO | 1.58 (1.24, 2.01) | <0.001 | 1.92 (1.46, 2.51) | <0.001 |
| CRAO | 1.01 (0.67, 1.54) | 0.946 | 1.08 (0.68, 1.73) | 0.742 |
| BRAO | 1.37 (0.81, 2.33) | 0.238 | 1.69 (0.94, 3.03) | 0.079 |
| TRAO | 2.23 (1.55, 3.20) | <0.001 | 2.94 (1.98, 4.36) | <0.001 |
| RVO | 1.42 (1.22, 1.65) | <0.001 | 1.77 (1.50, 2.09) | <0.001 |
| CRVO | 1.28 (1.01, 1.62) | 0.044 | 1.75 (1.34, 2.27) | <0.001 |
| BRVO | 1.47 (1.22, 1.76) | <0.001 | 1.70 (1.39, 2.09) | <0.001 |
| Age, 20-40 |  |  |  |  |
| Retinal vascular occlusion | 1.58 (1.27, 1.95) | <0.001 | 2.04 (1.62, 2.57) | <0.001 |
| RAO | 2.27 (1.59, 3.26) | <0.001 | 3.01 (2.04, 4.45) | <0.001 |
| CRAO | 1.85 (0.70, 4.86) | 0.214 | 1.71 (0.57, 5.10) | 0.338 |
| BRAO | 1.74 (0.65, 4.64) | 0.268 | 1.63 (0.53, 5.01) | 0.394 |
| TRAO | 2.51 (1.64, 3.83) | <0.001 | 3.54 (2.24, 5.57) | <0.001 |
| RVO | 1.27 (0.98, 1.66) | 0.073 | 1.58 (1.18, 2.10) | 0.002 |
| CRVO | 1.35 (0.89, 2.05) | 0.161 | 1.84 (1.17, 2.91) | 0.009 |
| BRVO | 1.18 (0.86, 1.62) | 0.313 | 1.38 (0.97, 1.96) | 0.075 |
| Age, 40-60 |  |  |  |  |
| Retinal vascular occlusion | 1.23 (1.11, 1.36) | <0.001 | 1.54 (1.37, 1.72) | <0.001 |
| RAO | 1.76 (1.45, 2.15) | <0.001 | 2.18 (1.76, 2.71) | <0.001 |
| CRAO | 1.04 (0.69, 1.58) | 0.847 | 1.32 (0.83, 2.10) | 0.239 |
| BRAO | 1.42 (0.88, 2.29) | 0.150 | 1.82 (1.07, 3.10) | 0.027 |
| TRAO | 2.09 (1.63, 2.69) | <0.001 | 2.57 (1.95, 3.38) | <0.001 |
| RVO | 1.07 (0.95, 1.20) | 0.256 | 1.33 (1.17, 1.52) | <0.001 |
| CRVO | 0.87 (0.70, 1.07) | 0.179 | 1.02 (0.81, 1.29) | 0.857 |
| BRVO | 1.18 (1.03, 1.35) | 0.018 | 1.46 (1.25, 1.69) | <0.001 |
| Age, 60-80 |  |  |  |  |
| Retinal vascular occlusion | 1.32 (1.18, 1.48) | <0.001 | 1.80 (1.59, 2.04) | <0.001 |
| RAO | 1.32 (1.04, 1.67) | 0.022 | 1.74 (1.34, 2.25) | <0.001 |
| CRAO | 1.05 (0.72, 1.54) | 0.787 | 1.24 (0.81, 1.90) | 0.316 |
| BRAO | 1.14 (0.71, 1.84) | 0.586 | 1.67 (0.98, 2.83) | 0.060 |
| TRAO | 1.88 (1.27, 2.79) | 0.002 | 2.55 (1.66, 3.91) | <0.001 |
| RVO | 1.31 (1.16, 1.49) | <0.001 | 1.78 (1.55, 2.05) | <0.001 |
| CRVO | 1.34 (1.09, 1.64) | 0.005 | 1.72 (1.38, 2.16) | <0.001 |
| BRVO | 1.28 (1.10, 1.49) | 0.001 | 1.75 (1.48, 2.07) | <0.001 |
| Age, ≥80 |  |  |  |  |
| Retinal vascular occlusion | 1.57 (1.03, 2.40) | 0.034 | 2.05 (1.28, 3.27) | 0.003 |
| RAO | 1.91 (0.78, 4.64) | 0.156 | 2.51 (0.97, 6.49) | 0.057 |
| CRAO | 2.10 (0.66, 6.71) | 0.210 | 2.92 (0.86, 9.94) | 0.086 |
| BRAO^b^ | - | - | - | - |
| TRAO | 2.39 (0.48, 11.89) | 0.286 | 4.46 (0.83, 23.8) | 0.080 |
| RVO | 1.47 (0.93, 2.33) | 0.102 | 1.83 (1.09, 3.09) | 0.022 |
| CRVO | 1.29 (0.66, 2.52) | 0.460 | 1.55 (0.72, 3.36) | 0.264 |
| BRVO | 1.41 (0.80, 2.51) | 0.238 | 1.67 (0.88, 3.16) | 0.116 |
| cHR: crude hazard ratio; aHR: adjusted hazard ratio; CI: confidence interval.  RAO: retinal artery occlusion; CRAO: central retinal artery occlusion; BRAO: branch retinal artery occlusion; TRAO: transient retinal artery occlusion; RVO: retinal vein occlusion; CRVO: central retinal vein occlusion; BRVO: branch retinal vein occlusion.  a: Adjusted HR estimated by the multivariable Cox proportional regression model including the variables of age, gender, and comorbidities.  b: n=0. | | | | |


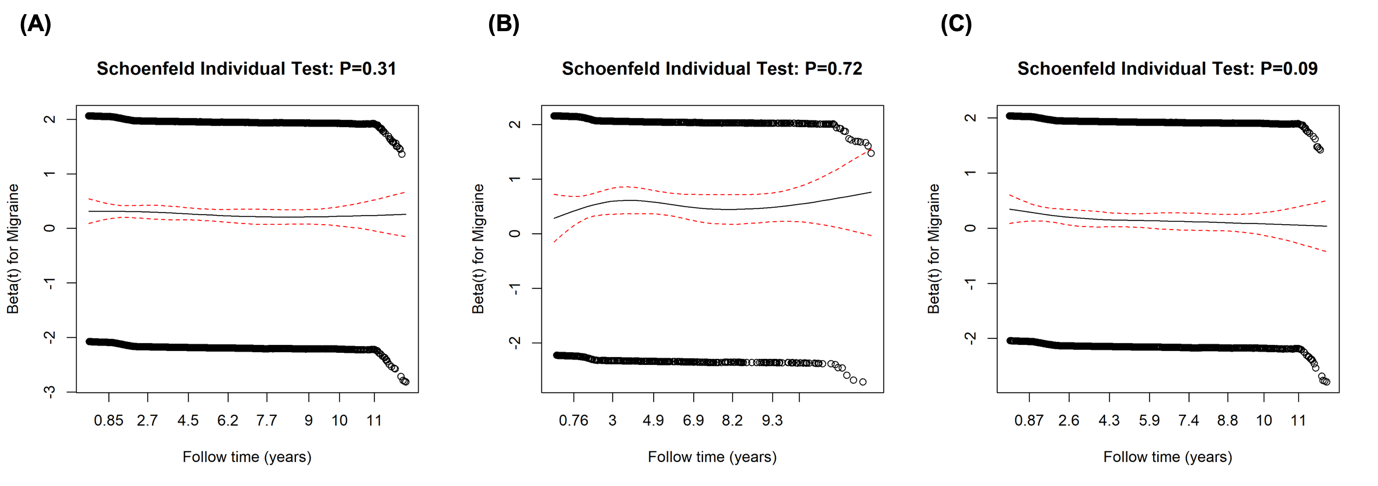


**Supplementary Figure S1.** The analyses of Schoenfeld residuals of (A) retinal vascular occlusion, (B) retinal artery occlusion (RAO) and (C) retinal vein occlusion (RVO).
